# Reconceptualising labour utilisation and underutilisation with new ‘full-time equivalent’ employment and unemployment rates

**DOI:** 10.1186/s12651-025-00396-z

**Published:** 2025-03-04

**Authors:** Donald Houston, Colin Lindsay

**Affiliations:** 1 City-Regional Economic Development Institute, University of Birmingham, Edgbaston, Birmingham, B15 2TT UK; 2 Department of Work, Employment and Organisation, University of Strathclyde, 199 Cathedral Street, Glasgow, G4 0QU UK

**Keywords:** Labour utilisation, Labour underutilisation, Unemployment, Underemployment, Gender, Youth unemployment, Regional unemployment, Labour market slack, Monetary policy, J64, R23

## Abstract

Time-related underemployment (wanting to work more hours) has become an entrenched feature of a number of mature economies since the Global Financial Crisis of 2008, recent short-run post-COVID labour shortages notwithstanding. Employment and unemployment rates are thus increasingly inadequate measures of labour utilisation and underutilisation. This paper develops novel ‘Full-Time Equivalent’ (FTE) employment and unemployment rates based on hours worked and hours wanted calibrated to a 37.5-h full-time week for the United Kingdom. FTE rates reveal greater labour market slack than evident in conventional measures, as well as lower utilisation and/or greater underutilisation among women, young people, low-skilled workers and in geographically and economically peripheral regions. The FTE employment rate shows statistically significant correlations with both earnings and labour demand across UK local labour markets, whereas the conventional employment rate fails to detect this relationship. The paper argues that the use of FTE metrics by policy makers would point towards, firstly, more demand-side labour market policies in weaker local labour markets rather than relying heavily on coercive supply-side labour market activation and, secondly, less hawkish monetary policy required to control inflation, which causes unnecessary harm to economically weaker regions.

## Introduction

Underemployment (people wanting to work more hours), overemployment (people wanting to work less hours), part-time employment and variable hours have become more prevalent in the labour market, particularly since the Global Financial Crisis of 2008. Yet existing headline labour market indicators published by most national and international statistics agencies are based on counts of persons rather than hours, in particular (a) the employment rate and (b) the unemployment rate. Published underemployment rates are based on the number of underemployed persons expressed as a proportion of all employed persons. It is therefore important that key widely-used labour market indicators are able to respond and adequately capture this fluidity and complexity. In order to better measure labour utilisation and underutilisation in the context of prevalent underemployment, overemployment and part-time and variable weekly hours, we have created hours-based equivalents of the employment rate and the unemployment rate, namely: the full-time equivalent employment rate (FTEER) and the full-time equivalent unemployment rate (FTEUR). The FTEER captures total hours worked, including overemployment. The FTEUR represents the hours wanted by the unemployment and the underemployed.

Labour utilisation and underutilisation are important for a range of purposes and policy areas, in particular labour market and monetary policies, making it important to have accurate measures. Labour utilisation is an important measure of the prevalence of employment as a source of income in a population, as well as an (inverse) indication of the level of potential labour for economic expansion (Topilin 2019; Fontanari et al. 2022). Labour underutilisation is an important measure of immediately available labour supply, so is important in macroeconomic management. Different demographic groups are more prone to different forms of involuntary worklessness, for example young workers are at greater risk of unemployment but it tends to be of shorter duration than for older workers (Axelrad et al. 2018), therefore rich and diverse measures of labour underutilisation are important in order to fully understand inequalities in the labour market. Labour utilisation and underutilisation and associated labour market policies display regional unevenness (Bacher et al. 2017), so are important indicators in regional development.

Time-related underemployment (wanting to work more hours), including the use of ‘zero-hours contracts’, has become an entrenched feature of the UK labour market since the Global Financial Crisis of 2008, and has risen in most mature economies (Walling and Clancy 2010; Bell and Blanchflower 2011, 2013, 2021; Hane-Weijman 2021; Rodríguez Hernández 2021). The high prevalence of underemployment and part-time working make conventional employment and unemployment rates increasingly inadequate measures of labour utilisation and underutilisation, respectively (Bell and Blanchflower 2014, 2018a, 2018b, 2021; Trapeznikova 2017; Fontanari et al. 2022). Specifically, the International Labour Office (ILO) definition of employment counts anyone working one or more hours per week as employed, not accounting for hours worked. The ILO unemployment rate does not capture those working but searching and available to work more hours—the underemployed (an exception is Germany where job seekers currently working up to 15 h per week are counted as unemployed rather than employed). Full-Time Equivalent (FTE) rates are not intended to imply that part-time employment is a bad thing, as a great many part-time workers do not want to work full-time. Rather, FTE rates are intended to be more comprehensive measures of labour utilisation and underutilisation than conventional employment and unemployment rates. The crucial issue is that the ILO employment rate over-estimates the true level of labour utilisation, and the ILO unemployment rate under-estimates the true level of labour underutilisation. Attempts have been made to integrate unemployment and underemployment into broader measures of labour underutilisation by simply summing counts of people ignoring the number of additional hours wanted and often adding “marginally attached” inactive workers (who want a job but are not search and/or available to start work).

This over-counting of labour utilisation has arguably (mis-)informed a dysfunctional active labour market policy regime and hawkish monetary policy in the UK and other mature capitalist economies for decades. Misleading measures of the true nature and extent of labour underutilisation partly explains why successive governments have failed to balance the supply-side and demand-side of labour market policy interventions (Ingold and McGurk 2023), been reluctant to make demands of either employers or activation providers in terms of decent work (Johnson et al. 2023), and have paid limited attention to solving the specific problem of underemployment (Houston et al. 2024).

Therefore, there is a need to integrate the unemployed and the additional hours wanted by the employed into a single measure that reflects the combined overall level of labour underutilisation to adequately reflect involuntary worklessness and available labour supply in the active workforce. We calculate such a measure, which we term the Full-Time Equivalent Unemployment Rate (FTEUR). Similarly, there is a need to calculate the rate of labour utilisation based on the number of hours worked rather than the number of persons employed in accurately measuring labour utilisation. We therefore calculate a novel FTE Employment Rate (FTEER) as the total number of hours worked per week divided by 37.5 * the working-age population. The FTEER advances the current state-of-the-art in hours-based measures of labour utilisation, currently expressed as average annual hours worked (Fontanari et al. 2022) by expressing it as a percentage rate. This makes it directly comparable to the conventional person-based employment rate, and is heuristically more intuitive to interpret than annual hours.

Our analysis is the first to assess social and spatial patterns in hours-based labour utilisation and underutilisation. It also provides a systematic comparison of the new FTE rates with conventional rates. We find considerable differences in the level of the FTE and conventional measures of both labour utilisation and underutilisation. We then compare FTE and conventional rates by gender, age, qualifications and region. The new FTE rates show some marked differences to conventional rates across these categories. Furthermore, FTE rates reveal spatial patterns of labour utilisation and underutilisation not detected in conventional rates for women, young people and low-skilled workers.

Finally, the paper goes on to assess the relationships of the new FTE measures’ with labour productivity and wage rates across local economies. This is the first paper to explore hours-based measures spatially, with previous work within economics examining relationships through the temporal dimension only. Our analysis demonstrates stronger relationships with these underlying economic indicators than those displayed by conventional rates. This demonstrates the robustness of the new FTE measures, with implications for the measurement of labour utilisation and underutilisation used in a range of policy areas.

As noted above, all of this matters because a broader and deeper understanding of labour underutilisation may direct us towards differing priorities for employment and regional development policies. Supply-side labour market policy under successive governments in many mature capitalist economies has prioritised ‘work-first’ activation of the unemployed and inactive with (until very recently) limited interest in incentivising individuals or employers to ensure that the former gain sufficient hours of work (Jones 2022). The rise in underemployment is part of wider polarisation and increasing non-standard employment in mature economies, including the UK (Green et al. 2016; Salvatori 2018) and Germany (Eichhorst and Tobsch 2015; Giannelli 2016).

Nor have issues of under-employment and working hours gained prominence in industrial and employment policy. The UK Government’s (2017: 22) short-lived Industrial Strategy committed government to working closely with sectors such as retail and tourism to “progressively drive up the earning power of people employed in these industries”, but offered no suggestions about how to respond to the high levels of under-employment (and more general problems of low pay) in these sectors. The issue of under-employment received a page in the UK Government-commissioned Taylor Review (2017) of workplace practices, but does not merit a specific policy recommendation within the same document. The more recent ‘Levelling Up’ White Paper makes vague assertions about the potential for new technology to drive better job quality, but again offers nothing on the sufficiency of income or work hours (UK Government 2022). The UK’s devolved administrations, especially Scotland and Wales, have more clearly articulated the need for workers to have security and sufficiency in hours as part of their ‘fair work’ agendas, but devolved administrations, like city-regions in England, arguably lack the policy levers to make a real difference in matters relating to employment (Houston et al. 2024). So, there is a need to provide a renewed focus on how public policy can step up to address underemployment and labour underutilisation. If we are to re-energise the policy debate around labour underutilisation, improving our measurement and understanding of the problem may prove an important first step.

## The measurement of labour utilisation and underutilisation

There are three main categories of labour market status, which are used for different purposes. The first is labour utilisation, chiefly captured in the employment rate, which is usually often used with the productive capacity in the economy, labour-market inclusion or poverty alleviation in mind. The second is underutilised labour, which can be thought of as involuntary worklessness or immediately available additional labour supply, often referred to as labour market slack. Underutilised labour is chiefly captured in the unemployment rate—defined as persons currently not working while searching and available to start work as a proportion of the workforce (the workforce comprising employed plus unemployed persons). Measures of labour underutilisation such as unemployment are used extensively in macroeconomic management, for example to avoid labour shortages and control wage inflation. A third, and rather heterogeneous, category is the economically inactive, which is those not employed and not searching and/or available to start work. Reasons for economic inactivity among the core working age (16–64 years) population include studying, looking after family and home, long-term sickness or disability and early retirement. A significant minority (typically around 20% of the inactive of working age) say they want a job, who can to some extent be considered as involuntarily out of work from a poverty alleviation and labour market exclusion point of view, but can less readily be seen as part of the active workforce (at least in the short run) from a labour supply point of view.

The new FTE rates reported in this paper relate only to the active workforce, i.e. the first two categories of economic activity—utilised and unutilised labour. The rationale for focusing only on the workforce and excluding the inactive who want a job (often referred to as the “marginally attached” or “hidden unemployed”) is for the first time to effectively integrate unemployment and underemployment into new single measures of labour utilisation and underutilisation that are expressed as a percentage rate and so can be directly compared to conventional employment and unemployment rates. The second reason to focus only on the active workforce is that several previous studies and national statistics agencies have developed various broader measures of labour underutilisation by summing various combinations of the unemployed, underemployed (often proxied by those involuntarily working part-time), discouraged workers and the marginally attached (or related measures of the hidden unemployed), but these suffer from neglecting to account for the number of hours worked and desired, i.e. they count people rather than hours.

*Utilised labour.* Rather than focussing on those not working, measures of labour utilisation relate to those who are working. A key measure of labour utilisation is the employment rate, or the proportion of the working age population who are employed—irrespective of hours worked. Following the definition of employment recommended by the UN’s International Labour Office (ILO), working only one hour per week is sufficient to be counted as employed, yet many want to work more hours—the underemployed. Other measures reported by national and international statistics agencies include: full-time employment rate; part-time employment rate; self-employment rate; and average hours worked. In Germany, the official unemployment rate includes people who are searching and available for work but currently working up to 15 h per week (Deutsche Bundesbank 2025), which serves to make the official German unemployment rate considerably higher than the ILO definition. Fontanari et al. (2022) develop an hours-based measure of labour utilisation for the USA, termed “standardized hours worked”, which is total aggregate annual hours worked divided by the working-age population (15–64 in the USA), which equates simply to average hours worked per person including the non-employed. The FTEER reported in this paper enhances this measure by expressing hours worked as a proportion (0–100%) of assumed normal full-time hours of 37.5 per week. This has the advantage of allowing direct comparison with the conventional person-based employment rate, and heuristically being more intuitive to interpret than annual hours.

*Underutilised labour.* Perhaps the most widely used measure of underutilised labour, which can be thought of as involuntary worklessness (from a social point of view) or available labour supply (from an economic point of view), is unemployment, defined by the ILO as searching and available for work while currently not working. Reflecting long-term rises in part-time employment, self-employment and casualization of employment (e.g. zero-hours contracts), increasing attention has been paid to the underemployed as a source of available labour supply. The ILO defines the underemployment rate as underemployed workers as a proportion of employed persons—with the underemployed defined, following the same principles of seeking and available in the definition of unemployment, as those currently employed but seeking and available to work more hours. This definition is followed by the UK’s Office for National Statistics (ONS) in its published underemployment rate (Walling and Clancy 2010; ONS 2022a). However, this measure fails to take account of the number of additional hours wanted by the underemployed, and it excludes the unemployed, therefore is an inaccurate and incomplete measure of the true level of labour underutilisation (Bell and Blanchflower 2011, 2013, 2014, 2018a, 2018b, 2021).

Several attempts have been made by scholars and national statistics agencies around the world to produce broader measures of labour underutilisation that add to the unemployed the underemployed and/or various groups of marginally attached inactive groups (see Olivieri and Paccagnella 2012). Although this approach has the benefit of creating a rate expressed as a percentage that is directly comparable to the conventional unemployment rate, it suffers from crudely summing workers with very different extents of labour utilisation/underutilisation, attaching equal weight to each. This ‘equal-weight’ problem is a common one in counting people who are in reality often simultaneously partly employed and partly unemployed. For example, Sibirskaya et al. (2021) add the unemployed, part-time workers and the marginally attached and express as a percentage of the total workforce defined in the same way. This is fine as a count of people experiencing some degree of involuntary worklessness or labour underutilisation, but is not an accurate measure of the overall aggregate level of underutilisation in the labour market. A perennial problem is that data are often not available on the number of additional hours wanted by the underemployed, or perhaps that counting people rather than hours is an engrained presumption in labour market indicators.

The US Bureau of Labor Statistics integrates unemployment and underemployment into its broadest “U-6” measure of unemployment by weighting each part-time worker who wants a full-time job at 0.5, but this is a very crude approximation of the actual number of additional hours wanted and excludes full-time workers who want to work more hours, stemming from the fact that ongoing survey data in the USA does not collect information on the number of additional hours wanted (Bell and Blanchflower 2021; ONS 2022b). A refinement is made by Komlos (2019) who weights involuntary part-time workers at 0.627, the ratio of average hours worked by part-time to full-time workers in the USA, and finds greater elevation in labour underutilisation when using this measure compared to conventional U-3 unemployment among ethnic minorities, young people and low-skilled workers. Faberman et al. (2020) develop what they term the Aggregate Hours Gap (between desired hours and actual hours worked) for the USA based on the difference between actual and desired hours of work from a job search survey conducted by the Federal Reserve Bank of New York, but again suffers from lack of national data on additional hours wanted in the USA.

Bell and Blanchflower (2013) develop for the UK what they term the “Bell and Blanchflower Underemployment Index”, which also integrates unemployment and underemployment, but enhances the “U-6” measure by capturing the number of additional hours the underemployed say they want to work, including full-time workers who want to increase hours (a problem with US data also identified by Addy et al. (2012)) and the assumed hours the unemployed will want to work on average. Unlike other broader measures of labour underutilisation that capture elements of “marginally attached” inactive persons, Bell and Blanchflower’s innovation is in restricting their index to the active workforce, i.e. only the unemployed and underemployed, with the latter following the ILO definition requiring active seeking of, and availability to start, additional hours of work. The beauty of this is that it provides a consistent and direct parallel with the job search and availability criteria to qualify as unemployed, producing a coherent and meaningful measure of labour market slack. In another divergence from other broader measures of labour underutilisation, Bell and Blanchflower (2014, 2018a, 2018b, 2019) argue that, in order to measure labour market slack for macroeconomic management, overemployment (those wanting to work less hours for less pay) needs to be subtracted to produce a ‘net’ underemployment figure, measured in hours. We do not share this view, on the basis that the overemployed are self-evidently currently willing and able to work hours in excess of their preferred hours—on the basis that they are already doing so. Crucially, the overemployed are not asked in the UK Annual Population Survey if they are actively seeking to reduce hours in their current job or by searching for a new job, therefore overemployment is not measured on a consistent basis with underemployment, with the latter requiring active seeking of additional hours. Furthermore, from a social point of view, someone else’s overemployment does nothing to lessen the impact of underemployment on the earnings or wellbeing of an underemployed person. Other attempts at measuring slack using hours-based measures have excluded overemployment (Faberman et al. 2020; Fontanari et al. 2022). We return to this issue in the Discussion and Conclusion section.

*Economic inactivity.* Following Bell and Blanchflower (2011, 2013, 2014, 2018a, 2018b, 2021) and Fontanari et al. (2022), this paper limits itself to producing new FTE indicators of labour utilisation and underutilisation among the economically active workforce. We provide a brief discussion here of involuntary worklessness among the economically inactive as important broader context. The economically inactive are defined as those not currently employed and either not searching for work and/or available to start work. Conversely, the economically active are either employed or unemployed (the latter defined as both searching and available for work). There has been much debate over many years as to what extent some members of the economically inactive population can be considered either potential labour supply and/or involuntarily not working, based variously on the concepts of discouraged workers, the hidden unemployed and the marginally attached.

Among the economically inactive, ‘discouraged’ workers are those who have concluded that searching for work is futile given their perceived low chances of securing work so are said to be inactive for economic reasons, and are included in the US Bureau of Labor Statistics “U-4” measure of unemployment. Discouraged workers do not meet the job seeking criterion to be considered unemployed, so are classified as economically inactive. Specifically, discouraged workers are defined as those not currently employed, want a job, are available to start work, but are not seeking a job because they think jobs are not available. According to this definition, discouraged workers only account for a tiny proportion of the UK’s economically inactive population aged 16–64—0.4% in the last quarter of 2019 immediately prior to the COVID-19 pandemic, falling to only 0.2% in the quarter to July 2022 (ONS 2022d).

A much higher proportion [22.1% in 2019 Q4 prior to the pandemic, falling to 19.2% in the quarter to July 2022 (ONS 2022d)] of the economically inactive population of working age say they want work, but may not be seeking and/or available to start work. The inactive who want a job are described as the “marginally attached” by the US Bureau of Labor Statistics and are included in its “U-5” and “U-6” measures of unemployment, reported in ONS (2022b). This group are sometimes termed the “hidden unemployed” (Baum et al. 2008). The desire for a job among the marginally attached or hidden unemployed may be somewhat hypothetical given their current circumstances may make this unrealistic (e.g. caring for family, studying, sick or disabled). All the inactive who want work cannot, therefore, be considered as part of the active workforce from a labour supply/macroeconomic management point of view, nor even fully involuntarily workless (otherwise they would be searching for work—with the possible exception of the very small group of discouraged workers). However, the fact that they want work helps capture the true level of labour market exclusion. Taking this broad definition of hidden unemployment, Baum and Mitchell (2010) calculate underutilisation rates for men and women in Australia by summing the unemployed and the hidden unemployed (similar to the United States’ “U-5” definition of unemployment), which has the effect of switching the greatest unemployment rate from men to women. However, limitations of this definition of underutilisation as the sum of unemployment and hidden unemployment is that it excludes the underemployed and attaches equal weight to those searching for work and those not searching.

Rather than counting all the inactive who say they want a job, using a more refined approach based on spatial patterns of labour market outcomes, Beatty et al. (2017) estimate that ‘hidden’ unemployment among recipients of disability-related state benefits across Great Britain contributes an additional 1.9% points to the unemployment rate, taking the ‘real’ level of unemployment from 3.8% to 5.7% of the working-age population in 2017. This estimate is based on the proportion of those in receipt of disability-related benefits that could be expected to be employed in conditions of near full-employment observed in some parts of the UK, taking account of the underlying poorer health of local populations in less prosperous districts, as fully elaborated in Beatty and Fothergill (2005).

### Labour utilisation and underutilisation in the UK

Before the COVID-19 pandemic, unemployment in the UK has fallen to one of its lowest historical levels, at 4.0% of the workforce in the last quarter of 2019 (ONS, 2022c). Similarly, the proportion of people of working age with a job—the employment rate—was at its highest ever (76.5%). However, 25.9% of employed people were working part-time and 7.7% of those employed were underemployed in the last quarter of 2019 (ONS, 2022a), while 2.6% were on zero-hours contracts in 2018 (ONS, 2019). Following the ILO’s definition of employment, any person working only one or more hours in the survey reference week will be counted as employed. Despite this, the employment rate remains a key headline reporting statistic, for example, “The employment rate (the proportion of people aged from 16 to 64 years who were in work) was estimated at 75.8%, higher than for a year earlier (75.2%) and *the joint-highest since comparable estimates began in 1971*” (ONS, 2019, emphasis added). Claims being made before the COVID-19 pandemic of recovery in the labour market following the Global Financial Crisis of 2008 and ensuing Great Recession, therefore, seemed premature with high levels of part-time working and underemployment among the employed, and 22.1% of economically inactive people of working age (16–64 years) in the last quarter of 2019 wanting a job (ONS, 2022d).

During the COVID-19 pandemic, employment in the UK fell from 33.0 million employed persons immediately prior to the pandemic to 32.1 million at the end of 2021, before recovering somewhat to 32.9 million in the Spring of 2022, still below its pre-pandemic level and falling again in the summer of 2022 following recruitment difficulties and sharp rises in inflation to stand at 32.7 million in the quarter to July 2022 (ONS 2022c). Part-time working fell by only one percentage point during the pandemic, from 25.9% of employed persons in the last quarter of 2019 to 24.9% in the quarter to July 2022. UK unemployment has fallen to 3.6% in the quarter to July 2022 (ONS, 2022c) after a surprisingly modest rise during social restrictions to control the COVID-19 pandemic in 2020 and 2021, in large part due to emergency employment protection put in place by the UK Government, termed the ‘furlough’ scheme. Underemployment tends to follow the same trends as unemployment (Bell and Blanchflower 2021) and so underemployment has correspondingly fallen since the pandemic to 6.6% of employed persons in the quarter to June 2022 (ONS 2022a). Nevertheless, even at a time of labour shortage and high job vacancies, the number of underemployed workers remains not far off double that of unemployment workers, and part-time working remains around a quarter of all employment. We do not wish to imply that part-time employment is a bad thing, as a great many part-time workers do not want to work full-time. Rather, the issue with part-time employment is that hours worked need to be accounted for in a precise measure of labour utilisation. Similarly, additional hours wanted need to be accounted for in a precise measure of labour underutilisation. Counting hours of work and desired additional work is more precise than counting people in an unweighted way.

## Data and method

### Analytical approach

In order to better measure labour utilisation and underutilisation in the context of prevalent underemployment and part-time employment, we have created hours-based equivalents of the conventional employment and unemployment rates for the UK using the three-year pooled Annual Population Survey 2016–18. We use the three-year pooled Annual Population Survey in order to generate a sufficient sample size to support an illustrative analysis of how the newly created FTE rates perform across 179 local labour markets. The Annual Population Survey and the three-year pooled Annual Population Survey are created by the UK’s Office for National Statistics through the aggregation and reweighting of relevant samples from the Quarterly Labour Force Survey. We name these new novel measures the full-time equivalent employment rate (FTEER) and the full-time equivalent unemployment rate (FTEUR).

Hours-based measures of the workforce (FTE employment and unemployment rates) work by reassigning underemployed hours among the employed from the employment rate to the unemployment rate. This has the effect of more accurately reflecting the aggregate level of labour utilisation (in the FTE employment rate) and labour underutilisation (in the FTE unemployment rate).

We compare conventional and FTE measures by gender, age, qualifications and region. This reveals lower utilisation in peripheral regions and considerable differences by gender, age and qualifications, which we then explore the geography of, and find that women and young people in particular display distinct geographies from all workers as a whole which are only revealed by the FTE measures.

We go on to conduct exploratory spatial analysis across local economies to assess whether our novel hours-based measures of labour utilisation (FTEER) and underutilised labour (FTEUR) have different relationships with underlying local economic conditions compared to conventional employment and unemployment rates, respectively. Our exploratory analysis is based on correlations between the local economic and labour market variables listed in Table 1, across the 179 NUTS3 regions of the UK. In order to run this analysis, we constructed a dataset for the 179 NUTS3 regions of key measures of underemployment, productivity and labour demand and supply (Table 1).

**Table 1:** Definition of measures and data sources

Measure	Source	Definition
*Person-based rates:*		
Employment rate	APS^1^ 2016–18	Employed persons as % of persons aged 16–64
Unemployment rate	APS^1^ 2016–18	Unemployed persons as % of economically active (employed + unemployed) persons
*Hours-based rates:*		
FTE employment rate	APS^1^ 2016–18	Total hours worked/37.5 as % of residents aged 16–64
FTE unemployment rate	APS^1^ 2016–18	Estimated hours wanted by the unemployed^5^ plus extra hours wanted by the employed as % of total potential hours (total potential hours = hours worked + extra hours wanted + estimated hours wanted by the unemployed)
*Local economic conditions:*		
Productivity	RPRD^2^ 2017	GVA per hour
Median hourly pay	ASHE^3^ 2017	GBP (£) per hour worked
25th percentile hourly pay	ASHE^3^ 2017	25th percentile pay per hour worked, GBP (£)
Job density	ONS Jobs Density^4^ 2017	Workplaces per resident aged 16–64

1—Authors’ calculations using 3-year pooled Annual Population Survey micro dataset January 2016 to December 2018; accessed via UK Data Service

2—ONS Regional Productivity Time Series (RPRD); GVA per hour reported in RPRD

3—Workplace-based hourly pay rates from the Annual Survey of Hours & Earnings (ASHE); accessed via Nomis

4—ONS Jobs Density data series comprising workplaces of employees, self-employed, government-supported trainees and HM Forces, as a proportion of residents aged 16–64 from ONS population estimates; accessed via Nomis

5—Mean hours wanted per unemployed person is assumed to be equal to mean hours worked + extra hours wanted by employed persons in each NUTS3 region

### Measuring labour utilisation and unutilised labour in local labour markets

Hours worked and hours wanted by the underemployed (nor even ONS’s simple underemployment rates) are not published for local areas or for key social breakdowns, for example age and gender. We therefore calculated conventional and FTE labour market indicators for key social categories and for local areas using the three-year pooled Annual Population Survey (APS) microdata for 2016–18. The advantage of the three-year pooled micro data is that it provides a sample size sufficient (n = 307,711 persons aged 16–64) to calculate rates for local areas not possible using annual or quarterly data (which do not include geographic identifiers below the 12 NUTS1 regions of the UK).

The only local identifier in the three-year pooled APS microdata available under ‘Safeguarded’ license for research use via the UK Data Service is EU NUTS3 regions, which are based on administrative areas of cohesive geographical, socio-economic, cultural, historic and environmental characteristics (European Union 2018). There were 179 NUTS3 regions across the UK, mostly corresponding to local government areas, or aggregations of local government areas, based on Unitary Authorities and local authority districts. In sparsely populated parts of Scotland, some geographically large local authority areas, e.g. the Highland council area, are broken into separate NUTS3 regions. The NUTS3 classification produces areas that are sufficiently small to capture contrasting local labour market conditions. NUTS3 regions offer an appropriate scale for the analysis of unemployment and underemployment, as they usually correspond to the spatial scale at which lower-skilled labour demand and supply matching takes place, display a good range of variation in employment outcomes (Kitsos and Bishop 2018).

## Results—contrasts between FTE (hours-based) and conventional (person-based) employment and unemployment rates

### Overall contrasts

By definition, the FTEER can only be less than or equal to the conventional employment rate; similarly, the FTEUR can only be greater than or equal to the conventional unemployment rate. What is striking is the size of the differences. The FTE hours-based employment rate for the UK 2016–18 was 11% points lower than the conventional person-based employment rate (63.6% versus 74.6%—Table 2). The UK’s FTE unemployment rate was double the conventional unemployment rate (9.1% versus 4.6%—Table 2). These are large differences.

**Table 2:** Comparison of conventional and Full-Time Equivalent employment and unemployment rates, by gender and age

Gender and age category	Full-time equivalent employment rate	Employment rate	*Diff*	Full-time equivalent unemployment rate	Unemployment rate	*Diff*
All (age 16–64)	63.6	74.6	*− 10.9*	9.1	4.6	*4.5*
Men	76.8	79.3	*− 2.6*	8.5	4.7	*3.9*
Women	50.7	69.9	*− 19.2*	9.9	4.5	*5.5*
Age 18–24	47.7	61.5	*− 13.8*	19.4	10.9	*8.5*
Age 25–49	73.6	83.5	*− 9.9*	7.7	3.4	*4.2*
Age 50–64	59.8	70.8	*− 11.0*	6.4	3.1	*3.2*

Source: Authors’ calculations using 3-year pooled Annual Population Survey micro dataset January 2016 to December 2018; accessed via UK Data Service; rates defined in Table 1; differences may not sum due to rounding

### Contrasts by gender, age and qualifications

Women and young workers display the greatest differences between person-based and hours-based measures of employment and unemployment (Table 2). On the FTE unemployment rate, women display greater unemployment than men—the opposite pattern to the conventional unemployment rate. This change in direction of gender imbalance in labour underutilisation when using hours-based rather than conventional person-based measures underlines the importance of developing and using alternative measures of labour utilisation and underutilisation.

The size of differences between person-based and hours-based measures for some groups typically disadvantaged in the labour market is remarkable. Women’s FTE employment rate is just under 51%, a whopping 19% points lower than the conventional employment rate of just under 70%. The FTE unemployment rate for younger workers (aged 18–24) is nearly 20%, almost double the conventional unemployment rate of just under 11%. The FTE unemployment rate for those with no qualifications is almost 15% (Table 3), 6% points greater than the conventional unemployment rate of just over 9%. The FTE employment rate for those with no qualifications is only 37% (Table 3). The sheer size of differences between conventional person-based and novel hours-based FTE measures, and the contrasts in the size of this difference between groups, reveal the true scale of inequalities in labour utilisation and underutilisation for some key groups disadvantaged in the labour market.

**Table 3:** Comparison of conventional and Full-Time Equivalent employment and unemployment rates, by highest qualification

Highest qualification	Full-time equivalent employment rate	Employment rate	*Diff*	Full-time equivalent unemployment rate	Unemployment rate	*Diff*
All	63.6	74.6	*− 10.9*	9.1	4.6	*4.5*
Degree +*	76.2	85.9	*− 9.7*	6.2	2.8	*3.4*
Other HE	68.9	80.9	*− 12.0*	7.4	3.0	*4.4*
GCE A level*	63.2	75.1	*− 11.9*	9.3	4.4	*4.9*
GCSE A*-C*	56.3	69.1	*− 12.8*	11.6	6.4	*5.2*
Other qual	59.5	69.2	*− 9.6*	12.7	6.6	*6.1*
No qualification	37.0	45.3	*− 8.3*	14.9	9.1	*5.7*

Source: Authors’ calculations using 3-year pooled Annual Population Survey micro dataset January 2016 to December 2018; accessed via UK Data Service; rates defined in Table 1; differences may not sum due to rounding

* or equivalent; GCE A Level = General Certificate of Education Advanced Level (post-16 years); GCSE A-C = General Certificate of Secondary Education (compulsory schooling to 16 years) top three grades (A-C)

In the next section, we explore how the geographical patterns of conventional person-based and novel hours-based FTE rates differ from each other. We do this for key groups identified above as having large overall national level differences; namely: women, young workers and those with no qualifications.

### The geography of FTE labour utilisation and underutilisation for key groups

Overall, both of the novel hours-based FTE measures display certain differences in their spatial pattern when compared to their conventional person-based equivalents, although the overall patterns are broadly similar (Table 4, All Workers). Specifically, the FTE employment rate lags the furthest behind the conventional employment rate in the far north and west (Scotland, South West and Wales). Although London displays the smallest gap between FTE and conventional employment rates, it is the only region to shift from below to above UK average when switching from the conventional to the FTE employment rate (Table 4, All Workers), reflecting more full-time jobs and longer hours worked in the capital city. London and Wales have the greatest gaps between conventional and FTE unemployment rates (Table 5, All Workers), with the highest recorded regional unemployment shifting from the North East to London when switching from the conventional to the FTE unemployment rate. Generally, FTE rates reveal that peripheral regions (in the north and west, further from the buoyant London and the South East) have weaker labour market outcomes than conventional rates imply—although the FTE unemployment rate also further reveals a high level of unemployment in London’s dynamic and competitive labour market. This finding with regard to peripheral regions is consistent with Olivieri and Paccagnella (2012) who found labour underutilisation was a greater problem in peripheral Southern Italy. Similarly, Tervo (1993) found large regional differences within Finland in hidden unemployment and underemployment. Hansen and Winther (2018) found peripherality was associated with slow employment recovery in Denmark following the Global Financial Crisis, although did not specifically investigate underemployment.

**Table 4:** FTE and conventional regional and urban–rural employment rates for key groups

Geographical area	All workers (16–64 years)			Women			Young workers (18–24 years)			Low-skilled workers (no qualifications)		
	FTEER	ER	*Diff*	FTEER	ER	*Diff*	FTEER	ER	*Diff*	FTEER	ER	*Diff*
UK	63.6	74.6	*− 10.9*	50.7	69.9	*− 19.2*	47.7	61.5	*− 13.8*	37.0	45.3	*− 8.3*
*Region*												
North East	58.9	70.5	*− 11.7*	48.1	66.9	*− 18.8*	43.9	58.4	*− 14.4*	32.1	40.1	*− 8.0*
North West	62.1	72.8	*− 10.8*	50.6	68.6	*− 18.0*	49.3	61.9	*− 12.7*	32.9	39.9	*− 6.9*
Yorkshire and The Humber	61.1	73.1	*− 12.0*	48.9	68.4	*− 19.5*	47.0	60.8	*− 13.8*	35.1	44.2	*− 9.0*
East Midlands	63.2	74.5	*− 11.3*	49.7	70.1	*− 20.4*	46.4	60.8	*− 14.3*	36.8	46.8	*− ****10.0***
West Midlands	62.0	72.4	*− 10.4*	48.3	66.8	*− 18.5*	45.0	57.7	*− 12.7*	36.9	45.4	*− 8.5*
East of England	**66.9**	**77.9**	*− 11.0*	**52.5**	73.3	*− 20.8*	**55.0**	**68.1**	*− 13.1*	**42.2**	**51.0**	*− 8.8*
London	66.0	74.1	*− 8.2*	**52.5**	67.6	*− 15.1*	44.2	54.1	*− 9.9*	35.3	44.1	*− 8.8*
South East	66.3	**77.9**	*− 11.6*	52.4	73.1	*− 20.7*	49.4	64.4	*− 15.0*	42.0	49.3	*− 7.3*
South West	65.3	78.0	***− 12.7***	51.5	**74.3**	*− ****22.8***	50.7	66.5	*− 15.7*	40.1	49.0	*− 8.9*
Northern Ireland	60.8	69.4	*− 8.7*	47.0	65.0	*− 17.9*	45.1	57.9	*− 12.8*	36.5	42.2	*− 5.8*
Scotland	61.8	73.9	*− 12.1*	50.3	70.1	*− 19.7*	47.9	65.8	*− ****18.0***	39.4	47.9	*− 8.5*
Wales	60.4	72.6	*− 12.1*	49.0	69.2	*− 20.2*	44.0	59.6	*− 15.7*	35.0	43.7	*− 8.8*
*Urban–rural classification*												
Predominantly urban	63.1	73.9	*− 10.8*	50.5	69.0	*− 18.6*	46.5	60.1	*− 13.6*	35.8	44.1	*− ****8.3***
Intermediate	**65.4**	**77.0**	*− ****11.6***	**51.6**	**73.0**	*− ****21.3***	51.6	**66.2**	*− ****14.5***	**41.2**	**49.4**	*− 8.2*
Predominantly rural	63.6	73.8	*− 10.2*	49.5	69.8	*− 20.4*	**51.9**	65.2	*− 13.3*	40.6	48.6	*− 8.0*

Source: Authors’ calculations using 3-year pooled Annual Population Survey micro dataset January 2016 to December 2018; accessed via UK Data Service; differences may not sum due to rounding; **greatest value (or largest absolute difference) in each column category in bold**; lowest value (or smallest absolute difference) in each column category underlined; FTEER = Full-Time Equivalent Employment Rate; ER = Employment Rate; rates defined in Table 1; Eurostat’s 2016 urban–rural classification of NUTS3 regions was matched by the authors to APS micro records

**Table 5:** FTE and conventional regional and urban–rural unemployment rates for key groups

Geographical area	All workers (16–64 years)			Women			Young workers (18–24 years)			Low-skilled workers (no qualifications)		
	FTEUR	UR	*Diff*	FTEUR	UR	*Diff*	FTEUR	UR	*Diff*	FTEUR	UR	*Diff*
UK	9.1	4.6	*4.5*	9.9	4.5	*5.5*	19.4	10.9	*8.5*	14.9	9.1	*5.7*
*Region*												
North East	10.2	**6.0**	*4.1*	10.5	**5.6**	*4.9*	21.9	13.1	*8.8*	14.9	10.8	*4.1*
North West	8.7	4.6	*4.1*	9.0	4.0	*4.9*	18.0	10.6	*7.4*	15.0	10.7	*4.3*
Yorkshire and The Humber	10.0	5.1	*5.0*	10.5	4.9	*5.6*	20.8	11.2	*9.6*	17.5	**12.7**	*4.8*
East Midlands	9.0	4.6	*4.4*	10.5	4.6	*6.0*	19.1	10.3	*8.8*	16.8	7.8	***8.9***
West Midlands	9.3	5.3	*4.0*	10.0	5.4	*4.6*	20.2	12.8	*7.4*	14.2	10.0	*4.2*
East of England	7.7	3.7	*4.0*	8.9	3.7	*5.2*	17.1	9.5	*7.6*	12.7	7.8	*4.8*
London	**10.5**	5.2	***5.3***	**11.7**	**5.6**	*6.1*	21.5	**13.4**	*8.2*	**17.6**	8.7	***8.9***
South East	8.1	3.7	*4.4*	9.3	3.8	*5.5*	18.7	9.8	*8.9*	12.3	6.9	*5.5*
South West	8.2	3.7	*4.5*	9.3	3.6	*5.8*	18.5	9.5	*9.0*	12.9	6.9	*6.0*
Northern Ireland	8.4	5.0	*3.3*	8.9	4.2	*4.6*	19.9	13.3	*6.6*	13.4	8.3	*5.1*
Scotland	9.2	4.4	*4.7*	9.5	4.0	*5.5*	17.7	8.0	*9.7*	13.8	8.4	*5.4*
Wales	10.1	4.8	***5.3***	11.0	4.7	***6.3***	**22.4**	12.2	***10.2***	16.6	10.1	*6.5*
*Urban–rural classification*												
Predominantly urban	**9.3**	**4.8**	*4.5*	10.1	**4.7**	*5.4*	**19.7**	**11.3**	*8.4*	**15.2**	**9.4**	*5.8*
Intermediate	8.4	3.9	*4.4*	9.4	3.7	*5.6*	18.5	9.6	***8.9***	14.5	8.5	***6.0***
Predominantly rural	8.9	4.1	***4.8***	**10.3**	4.0	***6.3***	17.8	9.8	*8.1*	11.8	8.1	*3.7*

Source: Authors’ calculations using 3-year pooled Annual Population Survey micro dataset January 2016 to December 2018; accessed via UK Data Service; differences may not sum due to rounding; **greatest value (or largest absolute difference) in each column category in bold**; lowest value (or smallest absolute difference) in each column category underlined; FTEUR = Full-Time Equivalent Unemployment Rate; UR = Unemployment Rate; rates defined in Table 1; Eurostat’s 2016 urban–rural classification of NUTS3 regions was matched by the authors to APS micro records

For women, the FTE rates reveal a different geography across urban–rural areas compared to conventional rates, which is not evident for All Workers (men and women combined). Specifically, women’s FTE employment rate is lowest in predominantly rural areas, but women’s conventional employment rate is lowest in predominantly urban areas (Table 4, Women). In contrast, switching from the conventional to the FTE employment rate has the opposite effect for All Workers (men and women combined), where the lowest conventional employment rate is in predominantly rural areas and the lowest FTE employment rate is in predominantly urban areas (Table 4, comparing Women and All Workers). Similar shifts in the urban–rural pattern of unemployment occurs for Women but not All Workers when switching from the conventional to the FTE rate (Table 5, comparing Women and All Workers). Thus, compared to all workers, the FTE rates reveal not only substantially different levels, but also different geographies, of employment and unemployment for women. Specifically, FTE rates reveal that women experience worse labour market outcomes in rural areas, but conventional rates mask this completely. These findings echo those of Baum et al. (2008, 2009) and Baum and Mitchell (2010) in relation to gender and metropolitan versus non-metropolitan patterns of underemployment in Australia.

For young workers, the FTE employment rate falls appreciably further below the conventional employment rate in Scotland than in any other region (Table 4 Young Workers). Similarly, Scotland’s pole position of having the lowest youth unemployment rate in the UK shifts to the East of England when we switch from the conventional to the FTE measure of unemployment. Wales displays the greatest shortfall between the conventional and FTE unemployment rates and records the highest FTE youth unemployment (22.4%) of all UK regions, switching from London (13.4%) on the conventional measure (Table 5, Young Workers).

For low-skilled workers, the FTE employment rate falls furthest below the conventional employment rate in the East Midlands, and shifts from above to below the UK average when switching from the conventional to the FTE employment rate (Table 4, Low-skilled Workers). A similar regional difference emerges when turning to unemployment among low-skilled workers, where the largest gap between FTE and conventional rates is in London and the East Midlands, both of which shift from well below to well above the UK average when switching from the conventional to the FTE measure of unemployment (Table 5, Low-skilled Workers). Thus, more so in London and the East Midlands than in other regions, the conventional measure of unemployment rate masks the true extent of unemployment among low-skilled workers. Low-skilled workers may be particularly disadvantaged in securing adequate hours of work in these regions due to dynamic and competitive labour markets and a greater prevalence of migrant workers willing to work long hours.

### Contrasts across local labour markets

We now turn to explore the contrasts between FTE and conventional measures across local labour markets. We have data for all 179 NUTS3 regions across the UK. This means our constructed NUTS3 dataset is not a sample. In this sense, the correlation coefficients between variables describe the *actual* relationship, i.e. are not based on a sample of localities. However, the underlying APS microdata used to construct the NUTS3 dataset is a sample of residential addresses, so is subject to sampling error. The calculated statistical confidence of the correlation coefficients provide an indication of the likely sensitivity to relatively small changes in the data.

**Fig. 1:**
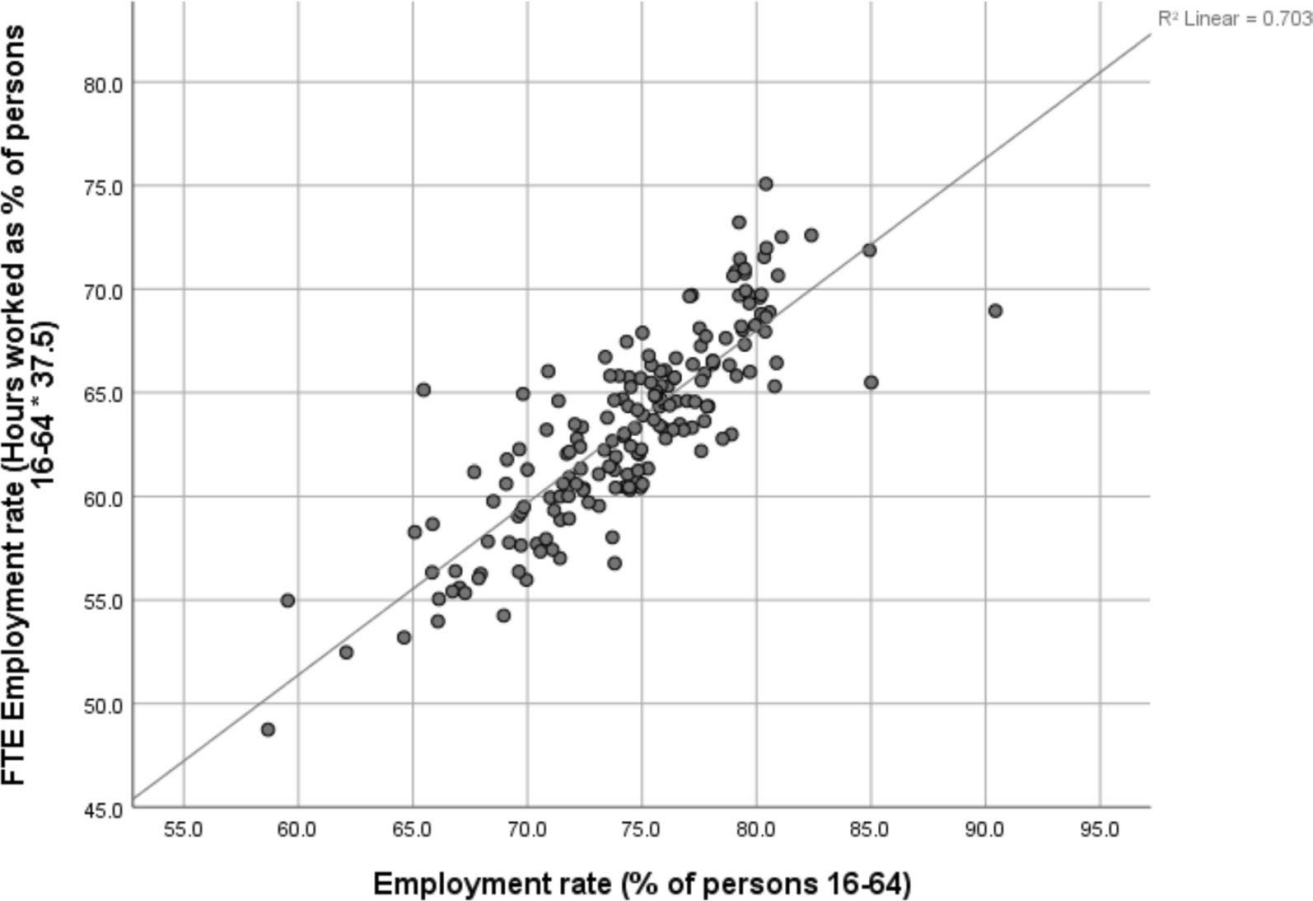
FTE employment rate and conventional employment rate, NUTS3 regions, 2017. Sources and definitions as set out in Table 1

**Fig. 2:**
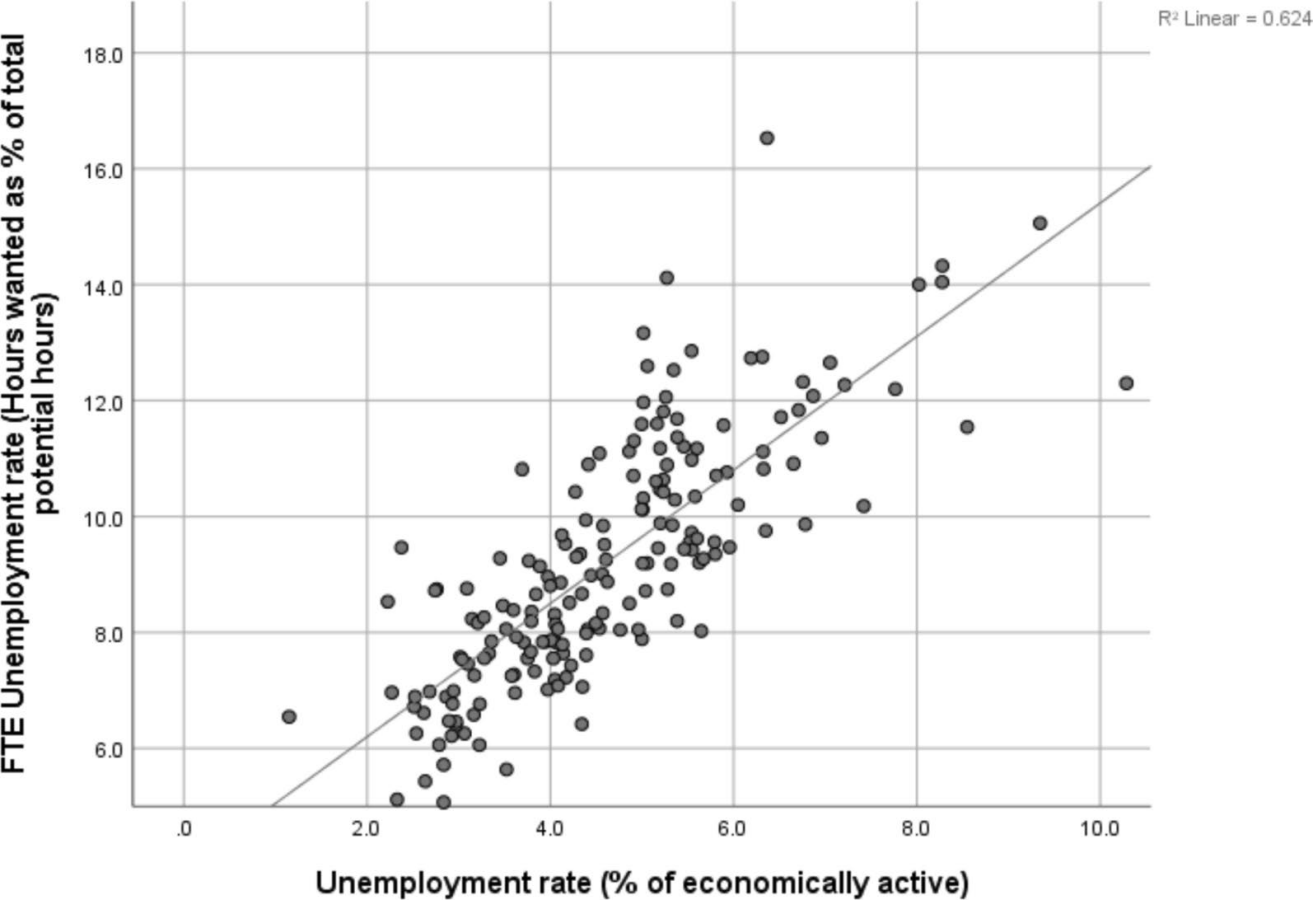
FTE unemployment rate and conventional unemployment rate, NUTS3 regions, 2017. Sources and definitions as set out in Table 1

There are relatively strong correlations between person-based and hours-based (expressed as FTE) measures of labour utilisation and underutilisation across NUTS3 regions (Figs. 1 and 2), although in some local labour markets the gap is larger than in others. The spatial pattern between the alternative measures differs more for unemployment (r = 0.790, Table 6) than for employment (r = 0.838, Table 6).

**Table 6:** Correlation coefficients between FTE and conventional measures of labour utilisation and underutilisation across local labour markets (NUTS3 regions)

	All workers (age 16–64)	Women	Young workers (age 18–24)	Low-skilled workers (no qualifications)
FTE/conventional employment rates	0.838	0.801	0.880	0.762
FTE/conventional unemployment rates	0.790	0.715	0.739	0.751

All signif > 99%

We extend the comparison of FTE and conventional rates across local labour markets to the groups examined in the previous sections. We do this by comparing the correlation between FTE and conventional rates for women, young workers and low-skilled workers against all workers (Table 6). The correlation between FTE and conventional rates is weaker for women, young workers and low-skilled workers than for All Workers, with the only exception of young workers’ employment rates. This general pattern of lower correlations between FTE/conventional across local labour markets for these disadvantaged groups indicates a greater difference in the spatial pattern of FTE and conventional rates for these three key groups of interest. Thus, FTE rates reveal a greater difference than conventional rates in the spatial pattern across local labour markets in labour market outcomes for women, young workers and low-skilled workers. The greatest divergence in spatial pattern between FTE and conventional rates is for unemployment among women (r = 0.715), followed by young workers (r = 0.739) and low-skilled workers (0.751). Low-skilled workers also have a relatively low correlation between the alternative measures of employment rate (r = 0.762).

### Exploratory analysis of relationships with local economic conditions

The aim of the exploratory spatial analysis is to assess whether our novel hours-based measures of labour utilisation (FTEER) and underutilisation (FTEUR) have different spatial relationships with underlying local economic conditions as provisional evidence that they more accurately measure labour utilisation and underutilisation. Our exploratory analysis is based on simple correlations between variables (Table 7). For analysis across NUTS3 regions, natural logs (ln) of economic variables (as indicated in Table 7) were taken in order to transform skewed distributions to enable parametric correlation analysis.

**Table 7:** Correlations coefficients across local economies of conventional and FTE measures of labour utilisation and underutilisation against local economic conditions

	ER	FTEER	UR	FTEUR
Labour productivity—GVA/hr (ln)	0.022	0.104	0.069	− 0.002
Median hourly pay (ln)	0.033	0.266**	0.073	0.034
25th percentile hourly pay (ln)	0.064	0.282**	0.053	0.050
Jobs Density (ln)	0.064	0.169*	− 0.007	− 0.070

*Signif > 95%; ** signif > 99%

The most striking finding is that the conventional employment rate is not correlated with either workplace hourly pay rates or with labour demand/jobs density, but the FTE employment rate is correlated with both (Table 7). This finding is true of both median and 25th percentile hourly pay rates. As a test for whether FTE rates have improved the measurement of labour utilisation, this suggests that the FTEER more accurately captures labour utilisation than the conventional employment rate. Crucially, the FTE employment rate reveals that labour utilisation is associated with pay and jobs density, which the conventional employment rate does not. Neither conventional nor FTE unemployment rates are correlated with workplace hourly pay rates or with jobs density. These findings are consistent with Fontanari et al. (2022)’s argument that it is preferable to measure hours-based utilisation rather than underutilisation because utilisation/employment avoids ambiguous assumptions about the hours wanted by the unemployed that arises in calculating an hours-based measure of underutilisation/unemployment. These findings also echo Dawkins and Wooden (1985) who argued that labour utilisation, rather than labour underutilisation, is central to understanding wage inflation.

## Discussion and conclusions

### The novelty of FTE employment and unemployment rates

As underemployment, part-time employment and variable hours become more prevalent in the labour market, it is important that the key widely-used labour market indicators are able to respond and adequately capture this fluidity and complexity. Existing headline labour market indicators published by national and international statistics agencies, including the UK’s ONS, are based on counts of persons, in particular (a) the employment rate and (b) the unemployment rate. Published underemployment rates are based on the number of underemployed persons expressed as a proportion of all employed persons. In order to better measure labour utilisation and underutilisation in the context of prevalent part-time and variable weekly hours, we have created hours-based equivalents of the employment rate and the unemployment rate, namely: the full-time equivalent employment rate and the full-time equivalent unemployment rate. FTE is assumed to be 37.5 h per week. FTE rates are intended to be more comprehensive measures of labour utilisation and underutilisation than conventional employment and unemployment rates. FTE rates are not intended to imply that part-time employment is a bad thing, as a great many part-time workers do not want to work full-time. Counting hours of work and desired additional work is more precise than counting people in an unweighted way.

The full-time equivalent employment rate captures the overall level of labour utilisation in hours per week worked expressed as a proportion of the working-age population * 37.5. The full-time equivalent unemployment rate integrates unemployment and underemployment into a single measure of the overall level of labour underutilisation, namely total hours wanted expressed as a proportion of total potential hours (hours worked plus additional hours wanted), limited to those actively seeking to increase their hours. The FTE unemployment rate does not require any assumption about ‘normal’ full-time hours because it is calculated solely using hours, although it does require the estimation of how many hours unemployed persons want to work.

Hours-based measures of labour utilisation and underutilisation (FTE employment and unemployment rates) work by reassigning underemployed hours among the employed from the employment rate to the unemployment rate. This has the effect of more accurately reflecting the aggregate level of utilised labour (in the FTE employment rate) and unutilised labour (in the FTE unemployment rate).

The FTEER advances the current state-of-the-art in hours-based measures of labour utilisation, currently expressed as average annual hours worked (Fontanari et al. 2022) by expressing it as a percentage rate. This makes it directly comparable to the conventional person-based employment rate, and is heuristically more intuitive to interpret than annual hours. The FTEUR differs from the Bell and Blanchflower Underemployment Index in that we do not subtract overemployment, following Faberman et al. (2020) and Fontanari et al. (2022) in order to produce a measure of the absolute level of labour underutilisation that treats underemployment in a manner that is consistent with the UN’s ILO definition of unemployment.

### Issues to consider in calculating and interpreting FTE employment and unemployment rates

A number of issues arise in the calculation and interpretation of FTE employment and unemployment rates. We discuss these issues here, mainly with a view to assisting those that may wish to develop FTE employment and unemployment rates for other countries or time periods. Three key issues and their implications are discussed: (i) what constitutes ‘normal’ full-time hours; (ii) how to deal with overemployment (people working more hours than they want); and (iii) data availability on additional hours wanted.

Firstly, for the FTE employment rate, an assumption needs to be made as to what constitutes ‘normal’ full-time hours. Note that this issue does not arise in relation to the FTE unemployment rate because it is calculated solely using extra hours wanted and hours worked in a labour force (the ratio of total extra hours wanted to total potential hours in the labour force, as defined in Table 1), avoiding the need to define ‘normal’ full-time hours.^1^ Since this paper uses UK data, the FTE employment rate has been calculated based on the typical contracted full-time hours in the UK of 37.5 h per week, although typical ‘full-time’ hours vary by occupation, industry and employer. Further ambiguity over how many hours constitutes full-time comes from flexibility over contracted hours in some sectors and offered by some employers making the notion of a singular ‘full-time’ contract ambiguous even within the same employer—for example in retail many employees are offered as many or as few shifts (or half shifts) as they wish, and this can be reflected in permanent contracts for some staff. ‘Normal’ full-time hours could be empirically defined, most obviously as the average actual hours worked by workers who describe themselves ‘full-time’. Average full-time hours worked, however, is likely to inflate what would be considered by most people to be ‘normal’ full-time hours on the basis that the average would include those working excessive hours, either voluntarily or involuntarily. Different countries have different prevailing norms around typical full-time working hours. Similarly, typical working hours have generally declined throughout industrial history driven by labour-saving technological advances and demands for more leisure time as incomes rise, making comparisons of FTE employment rates based on a fixed number of hours (e.g. 37.5 per week) over historic timespans problematic. Thus, international and temporal comparisons of FTE employment rates would need to be interpreted with caution (although the issue of international and temporal comparability of labour market indicators is not unique to our novel FTE employment rates).

The second issue that arises in calculating the FTE unemployment rate is whether to subtract overemployment (extra hours involuntarily worked by some employed persons) from the extra hours wanted. The resolution of this issue depends on what exactly is to be measured. Bell and Blanchflower’s Underemployment Index (which excludes unemployment) subtracts overemployment, but in this paper we do not. If seeking to measure how closely the level of labour utilisation matches that desired by the population, it makes sense to subtract overemployment in order to reflect the fact that some people would like to work more hours, counteracting (at population level, at least) that others would like to work less hours. However, if seeking to measure labour supply and potential labour supply, it does not make sense to subtract overemployment because, by definition, these hours are already being worked and therefore are logically part of labour supply. This paper is concerned with labour utilisation and underutilisation, therefore the FTE employment rate as specified in this paper includes overemployment in that all hours worked (including those that the overemployed would rather not work) are included in the calculation of the FTE employment rate. Similarly, unemployment (and less frequently underemployment) is usually used as a measure of labour supply or potential labour supply in macroeconomic management, which points towards the merit of not subtracting overemployment if a new headline FTE unemployment rate was to be created.

The third issue is the availability of data required to calculate FTE rates. The FTE employment rate is relatively straightforward in that it only requires data on the number of hours worked and the total population of working age. Even in the absence of a regular labour force survey, data on hours worked may be available from administrative sources (depending on data capture and availability in any given country, of course). The FTE unemployment rate is more demanding in terms of data, as it requires information on the number of additional hours wanted, including by the unemployed, which can only be obtained by asking people in surveys. In the UK Labour Force Survey, employed people have been asked every quarter since 1996 if they would like to work more hours and, if so, how many more (Office for National Statistics 2024).^2^ The unemployed are only asked if they are looking for full-time or part-time work, not precisely how many hours they want. Therefore, an assumption has to be made about how many hours an unemployed person, on average, wants to work. In this paper, we assumed this to be mean hours worked plus the extra hours wanted by employed persons. Employed persons are not representative of unemployed persons in terms of skills, age, occupation, etc., so this is a crude assumption, but a reasonable one for the purposes of the initial development of the FTE unemployment rate. Further research to refine the FTE unemployment rate could estimate hours wanted by the unemployed more precisely based on their traits in multivariate statistical models.

### What FTE employment and unemployment rates reveal and their policy implications

This paper has reported the first analysis (to our knowledge) of social and spatial patterns in hours-based measures of labour utilisation and underutilisation, as opposed to existing temporal analysis at national level. FTE rates reveal greater unemployment among women, the opposite gender pattern found in conventional unemployment rates. FTE rates reveal a much greater extent of labour market disadvantage for women, young workers and low-skilled workers than is apparent in conventional rates. We find that peripheral regions display the greatest gaps between conventional and FTE rates. London displays a greater gap between conventional and FTE unemployment rates, but a smaller gap between the alternative employment rates. The FTE measures display poorer labour market outcomes for women in rural areas, a regional difference masked in conventional rates. Scotland and Wales, but particularly Scotland, fare a lot worse for youth employment outcomes on the FTE measures than on conventional measures. One interpretation of this finding is that labour market inclusion policy initiatives in the devolved administrations of Scotland and Wales to support young people’s transition into employment have tended to shift people from unemployment into underemployment. The devolved administrations in both these territories have prioritised using policy levers (albeit limited given that employment is ‘reserved’ to the Westminster parliament) to promote ‘fair work’ (i.e. better job quality), but with relatively limited specific action on under-employment (Fair Work Commission 2019; Scottish Government 2022). Our findings suggest that a greater focus on under-employment may be justified in devolved administrations. Finally, FTE rates reveal that low-skilled workers’ poorer labour market outcomes are particularly pronounced in London and the East Midlands. These findings echo Perrons and Dunford (2013) who found different regional rankings within the UK for men and women on a composite regional development index, underlining the importance of gender-sensitive measures of regional inequality.

In terms of revealing relationships with underlying local economic conditions, a striking finding is that the conventional employment rate is not correlated with either workplace hourly pay rates or with labour demand/jobs density, but the FTE employment rate is correlated with both. These findings suggest that local economic growth is more important than labour market activation in raising labour utilisation. Furthermore, as a test for whether FTE rates have improved the measurement of labour utilisation, this suggests that the FTEER more accurately captures labour utilisation than the conventional employment rate, as we would a priori expect pay rates and jobs density to feed through to greater labour utilisation.

We are not implying that conventional person-based measures are flawed—simply that they measure different things to our newly created hours-based measures. Hours-based measures are a complement to, and not a replacement for, existing person-based measures. Existing conventional person-based measures capture the number and proportion of people who are employed and unemployed, which are crucial metrics in understanding labour market exclusion/inclusion. However, hours-based measures more accurately reflect aggregate levels of labour utilisation and underutilisation, which may be important in understanding the true extent of, and social, spatial and socio-spatial patterns in, employment and involuntary worklessness.

Conventional person-based labour market measures following ILO definitions of employment and unemployment overstate labour utilisation and understate underutilisation of available labour. Hours-based or ‘FTE’ alternatives more accurately capture aggregate labour utilisation and underutilisation, particularly in an era of widespread underemployment and part-time employment. A consequence is that there may be more productive potential and more labour market slack than is assumed in macro-economic management and monetary policy based on conventional measures of labour utilisation and underutilisation, and therefore monetary policy could afford to be less ‘hawkish’ when considering wage inflation pressures. A caveat is that some countries may not follow ILO definitions in all official measures of employment and unemployment rates. For example, in Germany a person searching and available for work but working up to 15 h per week is counted as registered unemployed, derived from eligibility rules for unemployment insurance (Deutsche Bundesbank 2025), but the same person would be counted as employed (unless they were working less than one hour per week) according to the ILO definition (which is also reported in Germany). Our conclusions are based on a comparison of our novel hours-based FTE rates relative to conventional ILO definitions, which are not necessarily followed in all official measures in all countries. Interestingly given its broader measure of registered unemployment, Germany has a relatively balanced suite of demand- and supply-side employment policies including strong regional policy (Martin et al. 2021), although the latter has of course been heavily shaped by reunification of East and West Germany in 1990.

Our conclusions also have important implications for labour market policy. Supply-side labour market policy in the UK has largely sought to “move people into any job as quickly as possible” irrespective of job quality (Lindsay et al. 2018: 318), which combined with welfare conditionality and harsh sanctions for those failing to seek any job irrespective of quality may have exacerbated problems of labour underutilisation in weaker labour markets. Policies to address concentrations of labour underutilisation and the negative consequences of under-employment need to grow beyond ‘work-first’ labour market activation and seek to engage employers in a commitment to deliver ‘fair work’ (including regular and sufficient hours) for those re-entering employment. If employers continue to resist, regulatory intervention would be justified. Kim and Golden (2022), reviewing the US context, have called for a strengthening of legislation enshrining a ‘right to request’ more hours and ‘access to hours’ regulations that (in some states of the US) require employers to demonstrate that additional contracted hours are offered to existing part-time staff before being advertised. Such minimal regulation could easily be applied were there the political will in the UK.

Finally, policymakers need to consider demand-side strategies for sectoral diversification in weaker labour markets and supply-side measures to upskill vulnerable workers as a route out of under-employing jobs and sectors (MacDonald 2019; Houston et al. 2024). In short, a range of demand-side and supply-side interventions could help mature economies to move beyond dysfunctional and ill-informed labour market policies that may have contributed to, rather than alleviated, labour underutilisation through excessive flexibilization and casualisation. Prior to any policy recalibration, however, comes work to better understand the parameters of the policy problem. We hope that our suggestions for reconceptualising labour under-utilisation are of value in understanding the prevalence of, and extent of regional unevenness in, this important challenge for labour market and monetary policies.

## Data Availability

The datasets analysed during the current study are available in: UK Data Service repository (Annual Population Survey Three-Year Pooled Dataset, January 2016—December 2018, Study Number SN8489), UK Office for National Statistics website (ONS Regional Productivity Time Series (RPRD)); and UK Office for National Statistics Nomis web database (Annual Survey of Hours & Earnings workplace analysis and Jobs Density). The composite dataset compiled from the above data sources for the correlation part of the analysis is available from the corresponding author on reasonable request.

## Footnotes

1 The astute reader will realise that, in principle, the FTE employment rate could be calculated using only hours worked and total potential hours, avoiding the need to define ‘normal’ full-time hours. However, this would be a different measure and not consistent with the conventional employment rate, which uses the entire population of working age (rather than the active labour force) as the denominator. This change in denominator would have the consequence that such a solely hours-based employment rate would complement the FTE unemployment rate to 1.0, i.e. the two would simply sum to 1.0 (or 100%), which contradicts how conventional employment and unemployment rates are calculated (they use different denominators and so do not merely complement each other to 100%). Conversely, the FTE unemployment rate calculated based solely on hours (as in this paper) *is* consistent with the conventional unemployment rate in that they both use the active labour force (or actively worked or sought hours, in the case of the FTE unemployment rate), rather than the entire population, as the denominator.

2 This means that the data are available to construct a time-series of hours-based FTE employment and unemployment rates on a quarterly or annual basis. The illustrative analysis in this paper, however, is concerned with a spatial analysis of how our novel FTE rates perform over contrasting local labour markets, which requires the larger sample of the three-year pooled Annual Population Survey.
